# Special Issue: “Spinal Deformity: Diagnosis, Complication and Treatment in Adolescent Patients”

**DOI:** 10.3390/jcm12020525

**Published:** 2023-01-09

**Authors:** Laura Scaramuzzo

**Affiliations:** Spine Surgery Division I, I.R.C.C.S. Orthopedic Institute Galeazzi, 20157 Milan, Italy; scaramuzzolaura@gmail.com

Adolescent idiopathic scoliosis (AIS) is a structural deformity of the spine involving all three planes of the space and arises in otherwise healthy children at or around puberty [1]. It is a progressive deformity with an established incidence of 2–4% around the world [1]. According to the Scoliosis Research Society (SRS) definition, AIS is diagnosed by the presence of a Cobb angle of a minimum 10° with a structural vertebral rotation [2]. The diagnosis of AIS is, generally, one of exclusion and made when congenital deformities, tumours, various neuromuscular syndromes or others subjacent pathologies have all been ruled out. The pathophysiology of AIS is still heavily debated in the medical literature. Several studies have identified a genetic aetiology, as patients with parents affected by AIS show an increased risk of developing deformity [3]. An important role is also played by alteration in oestrogens, as well as in calmodulin or melatonin levels, but vitamin D levels and low bone mineral density are also important predisposing factors [4]. Less serious curves presented a similar incidence among males and females; larger curves are more frequent in female patients [5]. Several aspects, such as skeletal maturity, sex, curve type, and magnitude at presentation, play a role in AIS development and progression.

The diagnosis of AIS is made both with clinical examination and specific radiological exams. The clinical evaluation includes a determination of the asymmetry of the shoulder and of the chest, and of pelvic obliquity, with the Adam forward-bending test used to detect the hump. The radiographic examination includes the use of a long cassette AP, an LL X-ray and a bending test to detect the curve flexibility. Additionally, whole-spine MRI is routinely used in the detection of AIS [6].

A delay in AIS treatment can be cause of faster progression of the deformity and consequent back pain, spinal decompensation, pulmonary function alterations and worsening of physical appearance [1]. Untreated AIS, at long-term follow-up, is responsible not only for physical but also for psychosocial diseases [2]. Curves of less than 30° showed a lower risk of progression in skeletally mature patients. Otherwise, curves between 30 and 50° have a higher risk of worsening during patient’s lifetime, generally by more than 10–15°. Curves over 50° have a progression of 1° per year [7].

There is no consensus on AIS treatment and there is a great variety of therapeutic strategies used in different countries. In skeletally immature patients, observation, followed by bracing if the curve progresses to greater than 25°, is the standard of care in North America. In Europe, especially in France, Germany and Spain, brace treatment is always accompanied by physical therapy [8]. Physical therapy is also the first conservative treatment for small curves and for low progressive curves [9]. The success of physical exercises or brace use in stopping the risk of curve development, improving deformity, or reducing the indications for surgery is still under debate, and no clear evidence to establish the best treatment for AIS has been found. Physiotherapy showed an important role in recent studies in preventing the development of the deformity in curves less than 25°, and also in improving the therapeutic effect of a brace, reducing the incidence of sequelae in curves between 25° and 45°. These results are reached by prescribing a combination of sport activities and kinesitherapy with the aim of improving the coordination, spinal proprioception, and movement control. The first goal of conservative treatment is the prevention of curve progression until the achievement of skeletal maturity, when the speed of progression of the curve dramatically decreases and consequently also the risk of surgery [1]. Otherwise, the indication for a surgical treatment of scoliosis in adolescent patients is taken into consideration for AIS with a Cobb angle of 40°–45°, or for patients in which a fast worsening of the pathology is expected. The purpose of surgical treatment is to achieve a three-dimensional correction of the affected segment of the spine while minimizing the number of fused levels [10]. A fundamental aspect of surgical AIS treatment is also the restoration of the sagittal balance; the lack of a proper sagittal balance can lead to flatback, back pain and progressive degenerative disk diseases in adult age [11]. A heated debate remains ongoing as to which surgical approaches are the most suitable for use in achieving the best deformity correction. The standard of care in AIS surgical treatment is to reach a balanced correction in all planes and to obtain a solid fusion in order to maintain a stable spine over time. AIS is a complex three-dimensional deformity involving both the spine and pelvis. These characteristics have been brought a rapid evolution of surgical instrumentations and techniques during the past fifty years, and three generations of instrumentation have succeeded since 1960s. Various systems have been used: hooks, pedicle screws and sublaminar wires, alone or together creating hybrid systems. All-hook constructs were considered the “gold standard” treatment for several years. In the last two decades, the pedicle screw implants have become increasingly popular thanks to their better biomechanical properties compared to hooks constructs [12]. The superiority of all-screws implants in scoliosis corrective surgery is now widely recognised. This has, also, influenced over the last years authors’ practice in adopting increasingly high-density constructs. [13]. Although implant density (ID) has been identified as a predictor of curve correction [14], the appropriate number of pedicle screws, required in order to obtain the best achievable and long-lasting correction, is still under debate. The massive insertion of pedicle screws, also, in the thoracic vertebrae for the correction of paediatric and adolescent deformities has been reported to be at low risk, also taking into account the high rate of screws malpositioning (11%) [15]. The high rate of screw malposition in AIS, superior also to that observed in adult patients, is probably due to the major incidence of smaller pedicles in these patients and of dysmorphism. The latter is especially prevalent in cases of thoracic AIS. In more recent years, to avoid possible neurological and vascular complications due to screw malpositioning, several technologies have been introduced with the aim of improving the accuracy of pedicle screw insertion. Examples of these include robotic-assisted pedicle screw placement, the use of intraoperative fluoroscopy and CT image-guided navigation, and 3D printed spine-models, the later typically being used for free-hand screw positioning in severe deformities. The introduction of technologies which help the surgeon to reduce the incidence of complications is very attractive and useful for the progression of the treatment of AIS.

Studies reported a complication rate of 6.3% for AIS surgery, with a mortality rate of 0.014 to 0.02 [15,16] The risk for perioperative and intraoperative complications is higherin most serious curves, in prolonged surgery and in combined approaches or when spinal osteotomies were included in the surgical planning [16].

The complexity of AIS aetiology, natural history and treatment widely, underlined in medical literature, should play a central role in surgeons’ indications and in the preoperative counselling with patients. Additionally, specific psychological and clinical support has to be provided to the patients during the entire treatment time.

In conclusion, many question remain still open on AIS aetiopathogenesis and natural history, a knowledge gap which inevitably negatively affect the possibility of a progression prediction and slows the development of targeted approaches. At the present day, the treatment of this disease has as its goal the prevention or correction of spinal deformities. More research is required to truly improve AIS prevention and management.

The issues raised in this editorial may help to give a clear view of the present situation of the spinal deformity pathology in adolescent patients and provide input for further innovations.

I would like to thank the reviewers in advance for their careful job and the *JCM* team for their precious help. Finally, a special thanks to the authors for their considerable contribution.

## References

[1] Weinstein S.L., Dolan L.A., Cheng J.C.Y., Danielsson A., Morcuende J.A. (2008). Adolescent idiopathic scoliosis. Lancet Lond Engl..

[2] Negrini S., Donzelli S., Aulisa A., Czaprowski D., Schreiber S., de Mauroy J., Diers H., Grivas T.B., Knott P., Kotwicki T. (2018). 2016 SOSORT guidelines: Orthopaedic and rehabilitation treatment of idiopathic scoliosis during growth. Scoliosis Spinal Disord..

[3] Kelly J., Shah N., Freetly T., Dekis J., Hariri O., Walker S., Borrelli J., Post N.H., Diebo B.G., Urban W.P. (2018). Treatment of adolescent idiopathic scoliosis and evaluation of the adolescent patient. Curr. Orthop. Pract..

[4] Addai D., Zarkos J., Bowey A.J. (2020). Current concepts in the diagnosis and management of adolescent idiopathic scoliosis. Child's Nerv. Syst..

[5] Konieczny M., Senyurt H., Krauspe R. (2013). Epidemiology of adolescent idiopathic scoliosis. J. Child. Orthop..

[6] Scaramuzzo L., Giudici F., Archetti M., Minoia L., Zagra A., Bongetta D. (2019). Clinical relevance of preoperative MRI in adolescent idiopathic scoliosis: Is hydromyelia a predictive factor of intraoperative electrophysiological monitoring alterations?. Clin Spine Surg..

[7] Reamy B.V., Slakey J.B. (2001). Adolescent idiopathic scoliosis: Review and current concepts. Am. Fam. Physician.

[8] Parent S., Newton P.O., Wenger D.R. (2005). Adolescent idiopathic scoliosis: Etiology, anatomy, natural history, and bracing. Instr. Course Lect..

[9] Negrini S., Aulisa L., Ferraro C., Fraschini P., Masiero S., Simonazzi P., Tedeschi C., Venturin A. (2005). Italian guidelines on rehabilitation treatment of adolescents with scoliosis or other spinal deformities. Eur. Med..

[10] Patel P.N., Upasani V.V., Bastrom T.P., Marks M.C., Pawelek J.B., Betz R.R., Lenke L.G., Newton P.O. (2008). Spontaneous lumbar curve correction in selective thoracic fusions of idiopathic scoliosis: A comparison of anterior and posterior approaches. Spine.

[11] Luk K.D.K., Vidyadhara S., Lu D.S., Wong Y.W., Cheung W.Y., Cheung K.M.C. (2010). Coupling between sagittal and frontal plane deformity correction in idiopathic thoracic scoliosis and its relationship with postoperative sagittal alignment. Spine.

[12] Liljenqvist U., Hackenberg L., Link T., Halm H. (2001). Pullout strength of pedicle screws versus pedicle and laminar hooks in the thoracic spine. Acta Orthop Belg..

[13] Bharucha N.J., Lonner B.S., Auerbach J.D., Kean K.E., Trobisch P.D. (2013). Low-Density versus high-density thoracic pedicle screw constructs in adolescent idiopathic scoliosis: Do more screws lead to a better outcome?. Spine J..

[14] Le Navéaux F., Aubin C.E., Larson A.N.L., Polly D., Baghdadi Y.M.K., Labetlle H. (2015). Implant distribution in surgically instrumented Lenke 1 adolescent idiopathic scoliosis. Spine.

[15] Hicks J.M., Singla A., Shen F.H., Arlet V. (2010). Complications of pedicle screw fixation in scoliosis surgery: A systematic review. Spine.

[16] Kwan M.K., Loh K.W., Chung W.H., Hasan M.S., Chan C.Y.W. (2021). Perioperative outcome and complications following single-staged posterior spinal fusion using pedicle scrw instrumentation in adolescent idiopathic scoliosis(AIS): A review of 1057 casa from a single centre. BMC Musculoskelet. Disord..

